# Thrombocytopenia in Pregnancy: A 5‐Year Analysis of Characteristics and Practices From a Tertiary Academic Center

**DOI:** 10.1111/ijlh.70187

**Published:** 2026-07-02

**Authors:** Mackenzie E. Lemieux, Ming Y. Lim

**Affiliations:** ^1^ Division of Internal Medicine University of Utah Salt Lake City Utah USA; ^2^ Division of Hematology and Hematological Malignancies, Huntsman Cancer Institute University of Utah Salt Lake City Utah USA

**Keywords:** gestational, immune thrombocytopenia, neuraxial anesthesia, pregnancy

## Abstract

**Background:**

Moderate‐to‐severe thrombocytopenia (platelet count < 100 × 10^9^/L) occurs in fewer than 1% of pregnancies, posing management challenges, particularly surrounding eligibility for neuraxial anesthesia. Although recent anesthesia guidelines recommend a platelet threshold ≥ 70 × 10^9^/L, outcomes data applying these recommendations in moderate‐to‐severe thrombocytopenia remain limited.

**Methods:**

We conducted a retrospective study of 306 pregnancy encounters at a tertiary U.S. center (January 2018–December 2022) with ≥ 1 documented platelet count < 100 × 10^9^/L. Etiology, platelet nadir, hematology consultation, treatment patterns, and neuraxial anesthesia (NA) use were assessed from antepartum through postpartum discharge.

**Results:**

Gestational thrombocytopenia (gTCP) was the most common etiology (29%, *n* = 92). Thrombocytopenia severity differed across etiology, with higher platelet nadirs in gTCP (mean 84, median 88.5 × 10^9^/L) compared with ITP (mean 62, median 66 × 10^9^/L). Overall, 15% of pregnancies received hematology consultation, the majority of which were for individuals with ITP, and 78% underwent NA. Among pregnancies complicated by ITP, 71% received NA. Hematology consultation in ITP was associated with lower platelet nadirs and higher treatment rates.

**Conclusions:**

In this cohort of moderate‐to‐severe thrombocytopenia, institutional adherence to guideline‐recommended platelet thresholds was high and associated with excellent neuraxial safety outcomes. These findings provide real‐world support for current anesthesia recommendations in a higher‐risk obstetric population.

## 1 Introduction

Thrombocytopenia, defined as a platelet count < 150 × 10^9^/L, complicates up to 10% of pregnancies and is usually mild [1]. However, moderate‐to‐severe thrombocytopenia (platelet count < 100 × 10^9^/L) occurs in fewer than 1% of pregnancies and poses increased risk for hemorrhage, caesarean sections, preterm delivery, and intrauterine fetal demise [2, 3, 4]. Moderate‐to‐severe thrombocytopenia also poses management challenges, particularly surrounding eligibility for neuraxial anesthesia. The Society for Obstetric Anesthesia and Perinatology (SOAP) recommends a platelet threshold of at least 70 × 10^9^/L for neuraxial procedures (Class IIa, Level C‐LD) [5]. However, published data describing real‐world practice patterns and outcomes following these guideline updates are sparse, with only one retrospective study specifically evaluating implementation and outcomes [6]. Real‐world data on guideline implementation are essential to inform larger‐scale studies and refine clinical practice guidelines.

We therefore conducted a 5‐year retrospective study to characterize the etiologies, management patterns, and neuraxial anesthesia outcomes among pregnancies complicated by moderate‐to‐severe thrombocytopenia at a tertiary academic medical center.

## 2 Methods

A retrospective review of pregnancies was conducted in women of childbearing age ≥ 18 years who had at least one live birth or fetal death (> 20 weeks of gestation) at the University of Utah Health, a tertiary academic medical center. Pregnancy encounters between January 1, 2018, and December 31, 2022, were determined using ICD‐10 diagnosis codes (Figure 1, Table S1).

**FIGURE 1:**
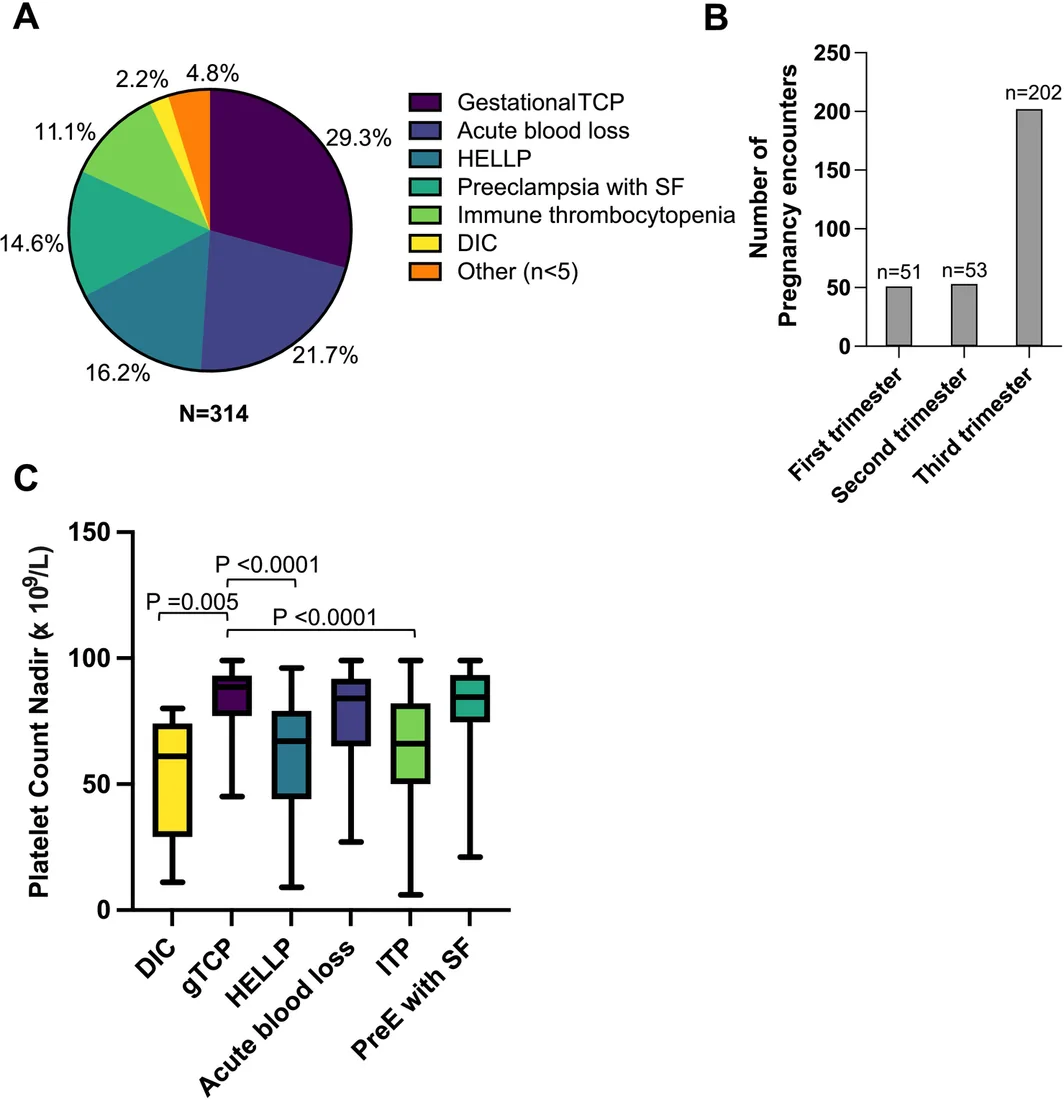
(A) pie chart depicting the percentage of each etiology of moderate to severe thrombocytopenia (< 100 × 10^9^/L) in our cohort (*N* = 314). Eight individuals had both gTCP and acute blood loss, so 314 diagnoses were included across 306 individuals. For rare diagnoses (< 5 pregnancy encounters), we created an “other” category for the purposes of figures and statistical analysis. Other includes: cirrhosis, aplastic anemia, von Willebrand disease, acute lymphocytic leukemia, acute promyelocytic leukemia, autosomal dominant macrothrombocytopenia, hepatitis, pseudothrombocytopenia, myelodysplastic syndrome, thrombotic thrombocytopenic microangiopathy. (B) bar chart depicting the number of pregnancy encounters on the *Y* axis and the trimester when moderate to severe thrombocytopenia was first identified on the *X* axis. First trimester = 0 weeks 0 days to 13 weeks 6 days; second trimester = 14 weeks 0 days to 27 weeks 6 days; third trimester = 28 weeks 0 days to delivery. (C) box and whisker plots depicting the median and IQR of platelet count nadirs for each etiology of moderate to severe thrombocytopenia. Whiskers correspond to the minimum and maximum platelet counts within each category. DIC = disseminated intravascular coagulopathy, gTCP = gestational thrombocytopenia, ITP = immune thrombocytopenia, PreE with SF = preeclampsia with severe features.

Pregnancy encounters with at least one documented platelet count of < 150 × 10^9^/L during the antepartum period through postpartum discharge were identified. Platelet counts were obtained from routine clinical complete blood counts performed in the University of Utah Health clinical laboratory using the Sysmex XN‐9000 model. Platelets were measured using impedance, with instrument‐based reflex to fluorescence if the platelet count was < 30 × 10^9^/L. Alternative counting was performed according to institutional laboratory protocols when the analyzer flags suggested platelet clumping or other potential analytic interference.

A total of 1203 unique pregnancies were identified, with some individuals contributing to > 1 pregnancy during the study period. Inclusion criteria were then narrowed to only include moderate to severe thrombocytopenia (platelet count < 100 × 10^9^/L). The following data were extracted: underlying cause and timing of thrombocytopenia in relation to gestational age, the occurrence and timing of hematology consultation (if any), the clinical management of thrombocytopenia (if any), the gestation and timing of delivery, and whether neuraxial anesthesia was permissible at delivery.

To determine the cause of thrombocytopenia, all documented peripartum and postpartum platelet counts were reviewed. The platelet nadir was used to determine the severity of thrombocytopenia and to determine the etiology when the nadir occurred. For individuals in whom the cause of thrombocytopenia was unclear due to no recorded laboratory values in the postpartum period or no possible causes identified via chart review, we listed the cause as gestational thrombocytopenia (gTCP).

A detailed review of the clinical management and outcomes for individuals with immune thrombocytopenia (ITP) was conducted, recognizing that unlike preeclampsia with severe features (PreE with SF), hemolysis, elevated liver enzymes, low platelets (HELLP), and gTCP, ITP has specific antepartum treatment options. Data on ITP‐directed therapies were collected, including the use of corticosteroids, intravenous immune globulin (IVIG), immunosuppressive agents, and platelet transfusions.

For statistical analysis, continuous variables were summarized using medians with ranges or interquartile ranges, and categorical variables were summarized as counts and percentages. Platelet nadirs were compared across etiologies using the Kruskal‐Wallis test followed by Dunn's multiple‐comparisons test. All statistical tests were two‐sided, and *p* < 0.05 was considered statistically significant. Analyses were performed using GraphPad Prism version 10.

This study was reviewed by the University of Utah Institutional Review Board and determined to be exempt under Category 4 for secondary research using existing clinical data. The requirement for informed consent was waived because the study involved retrospective review of existing records, posed minimal risk to participants, and data were analyzed in a de‐identified/limited dataset format. Data were stored on secure institutional servers in accordance with institutional privacy policies.

## 3 Results

Across 22 496 pregnancies over a 5‐year period, 5.3% (*n* = 1203) had at least one documented platelet count of < 150 × 10^9^/L during the antepartum period through delivery discharge (Figure S1). Of these, 25.4% (*n* = 306 pregnancies among 295 unique individuals) had at least one documented platelet count of < 100 × 10^9^/L (1.4% of all pregnancies) and were included for analysis in this study.

### 3.1 Demographics and Clinical Characteristics

Of the 306 pregnancy encounters affected by moderate‐to‐severe thrombocytopenia, the median age was 31 years (range: 18–48). The overall median platelet nadir was 80 × 10^9^/L (range: 6–99). Median gestational age at delivery was 37 weeks (range: 21–41). Overall, 15% of pregnancies (*n* = 45) received a hematology consultation, and 78% (*n* = 239) had neuraxial anesthesia (Table 1).

**TABLE 1:** *N*, distributions of platelet nadirs, age, hematology consultation status, presence of epidural, median gestational age across each etiology of thrombocytopenia (HELLP, hemolysis, elevated liver enzymes, low platelets; Preeclampsia with SF, preeclampsia with severe features; DIC, disseminated intravascular coagulation; GA, gestational age).

Etiology of thrombocytopenia	*N*	Mean platelet nadir (× 10^9^/L)	Median platelet nadir (× 10^9^/L)	Platelet range (× 10^9^/L)	Median age (years)	Hematology consultation (*n*, % yes)	Neuraxial anesthesia (*n*, % yes)	Median GA at delivery (weeks)
Overall^a^	306	87	80	6–99	31	45 (15)	239 (78)	37
Gestational Thrombocytopenia	92	84	88.5	45–99	32	10 (10.9)	80 (87)	39
HELLP	51	63	67	9–96	31	2 (4)	31 (61)	34
Immune Thrombocytopenia	35	62	66	6–99	29	17 (49)	25 (71)	38.5
Acute blood loss	68	77	84	27–99	32.5	4 (6)	58 (85)	38.4
DIC	7	54	61	11–80	27	0 (0)	5 (71)	35.3
Preeclampsia with SF	46	82	84.5	21–99	30.5	4 (9)	38 (83)	34.2

^a^ Overall total pregnancy encounters = 306, overall total unique individuals = 295, overall total diagnoses = 314. Overall calculation computed based on 306 total pregnancy encounters.

Nearly all pregnancy encounters affected by moderate‐to‐severe thrombocytopenia were attributable to a single underlying cause. Eight pregnancy encounters had two contributing diagnoses (e.g., gTCP and hemorrhage), and both were recorded, resulting in a total of 314 documented causes of thrombocytopenia, as shown in Figure 1A.

Of the 314 documented cases, gTCP was the most common cause of moderate‐to‐severe thrombocytopenia (29%, *n* = 92) (Figure 1A). ITP accounted for 11.1% (*n* = 35) of pregnancies and was the fifth most common diagnosis. One individual with a single platelet value of 86 × 10^9^/L at delivery and no alternative explanation was classified as gTCP.

Platelet nadirs differed significantly across thrombocytopenia etiologies (Table 1). Individuals with gTCP had higher platelet nadirs (mean 84, median 88.5 × 10^9^/L) than those with ITP (mean 62, median 66 × 10^9^/L). gTCP nadirs were significantly higher than HELLP (*p* < 0.0001), ITP (*p* < 0.0001), and disseminated intravascular coagulation (DIC) (*p* = 0.0055). Platelet nadirs were also higher in PreE with SF compared with HELLP (*p* = 0.0001) and DIC (*p* = 0.0257), and higher in acute blood loss compared with HELLP (*p* = 0.0051) (Figure 1C).

Most pregnancy encounters (*n* = 202, 66%) were first identified with moderate‐to‐severe thrombocytopenia in the third trimester (Figure 1B, total *n* = 306 pregnancy encounters). Nearly all deliveries (*n* = 288, 94%) occurred in the third trimester, and 61.4% (*n* = 188) were at term (> 36 weeks and 6 days).

### 3.2 Institutional Management Patterns for Moderate to Severe Thrombocytopenia

Hematology consultation rates were overall low (15% of pregnancies) and varied by etiology. The rates were highest among those presenting with ITP (49%, *n* = 17), followed by gTCP (11%, *n* = 10) and PreE with SF (9%, *n* = 4). Neuraxial anesthesia use was most frequent in gTCP (87%) and least frequent in HELLP syndrome (61%) (Table 1). Median gestational age at delivery also differed by etiology, with the earliest deliveries observed in HELLP syndrome (34 weeks) and PreE with SF (34.2 weeks), and the latest in gTCP (39 weeks).

One neuraxial procedure was performed at a platelet count of 46 × 10^9^/L, below the recommended threshold. This occurred when epidural placement preceded the availability of admission laboratory results in an individual with HELLP syndrome and previously normal platelet counts. All other neuraxial procedures were performed at a platelet count ≥ 70 × 10^9^/L. No epidural hematomas occurred following neuraxial anesthesia placement or removal.

Cesarean delivery occurred in 46.7% (*n* = 143) of deliveries. General anesthesia was documented in 21% (*n* = 30) of cesarean deliveries, of which four deliveries required conversion to general anesthesia after successful delivery of the fetus under regional anesthesia for intra‐operative complications or placenta accreta spectrum. Among vaginal deliveries (*n* = 163), 74.8% (*n* = 122) had neuraxial anesthesia, 17.2% (*n* = 28) had no analgesia documented, 4.9% (*n* = 8) had IV pain management, and 3.8% (*n* = 5) had nitrous oxide (Table S2).

### 3.3 Pregnancy Encounters Affected by Immune Thrombocytopenia

Among 35 pregnancy encounters with ITP, the median platelet nadir was 66 × 10^9^/L (range 6–99). Neuraxial anesthesia was performed in 71% (*n* = 25) (Table 1). Median platelet nadir was higher in those who received neuraxial anesthesia (70 × 10^9^/L) compared with those who did not (45 × 10^9^/L) (Table 2).

**TABLE 2:** Depicting the characteristics of individuals with immune thrombocytopenia (*N* = 35) by whether a hematology consult occurred and whether the individual received neuraxial anesthesia.

Variable	Hematology consult (yes), *n* = 17	Hematology consult (no), *n* = 18	Neuraxial anesthesia (yes), *n* = 25	Neuraxial anesthesia (no), *n* = 10
Platelet nadir, median (range)	53 (6–92)	78 (8–99)	70 (17–99)	45 (6–92)
Treated for ITP, *n* (%)	13 (76)	4 (22)	10 (40)	7 (70)
Steroids used, *n* (%)	12 (70)	4 (22)	9 (36)	7 (70)
IVIG used, *n* (%)	6 (35)	1 (5)	3 (12)	4 (40)
Platelet transfusion *n* (%)	1 (6)	2 (11)	1 (4)	2 (20)
GA at nadir, median (IQR)	35	37.5	37	34
Hematology consult, *n* (%)			10 (40)	7 (70)
Neuraxial anesthesia (yes) *n* (%)	10 (59)	15 (83)		

Hematology consultation occurred in 49% (*n* = 17), and 76% (*n* = 13) of those individuals received ITP‐directed therapy (Table 2). Consultation timing was most often antepartum (*n* = 13), with fewer consultations intrapartum (*n* = 2) or postpartum (*n* = 2) (Table 3). Five treated individuals had only partial treatment responses with final platelet counts < 70 × 10^9^/L and did not receive neuraxial anesthesia. Treatment initiation among these partial responders occurred primarily in the third trimester (Table 3). Treatment without hematology consultation occurred in four individuals. Median gestational age at platelet nadir was earlier among those who received hematology consultation (35 weeks) compared with those who did not (37.5 weeks) (Table 2).

**TABLE 3:** Individual patient‐level information for all individuals in our dataset with immune thrombocytopenia, with information about their treatment, timing of diagnosis, epidural status, presence of hematology consult, and platelet nadir.

Patient	Pregnancy	Gestation at delivery	Age	Secondary cause of TCP	Epidural	Hematology consult	Medications for ITP	Platelet nadir	Week of platelet nadir
1	1	39 weeks +1 day	31		Yes (platelets > 70)	No	No	83	39 weeks 1 day
2	1	40 weeks +3 day	28		Yes (platelets > 70)	No	No	85	40 weeks +3 days
3	1	38 weeks +1 day	38	Pre‐eclampsia	No	Yes, post‐partum	Dex 40 mg × 4 days, started post‐partum	41	38 weeks
4	1	33 weeks +6 days	19	Pre‐eclampsia	Yes (platelets > 70)	No	No	70	13 weeks +1 day
5	1	38 weeks +0 day	20		Yes (platelets > 70)	Yes, antepartum	Dex at 34 weeks +3 days, o response, then IVIG, with response	18	36 weeks +3 days
6	1	38 weeks	33		Yes (platelets > 70)	Yes, when admitted for delivery	No	88	38 weeks
7	1	35 weeks +1 day	40		No	Yes, antepartum	Pred 10 mg qD to 20 mg BID, started in first trimester and continued	52	She was on steroids throughout pregnancy
8	1	39 weeks +1 day	26		No	No	Dex 4 mg × 4 days at 38 weeks, no response, then IVIG + platelets (at delivery)	8	Was < 30 throughout pregnancy
9	1	36 weeks +4 day	29		Yes (platelets > 70)	No	No	95	29 weeks +3 days
10	1	38 weeks +6 day	30		Yes (platelets > 70)	Yes, antepartum	Pred 60 mg × 7 days, 40 mg × 4 days, and 20 mg × 3 days at 37 weeks GA	75	33 weeks of gestation
11	1	39 weeks +1 day	32		Yes (platelets between 96 and 62)	Yes, antepartum	Pred 60 mg × 7 days, 40 mg × 3 days, 20 mg × 3 days, at 37 weeks GA, then again postpartum for epidural removal	62	At delivery
12	1	35 weeks	19		No	Yes, antepartum	Pred 60 mg daily at 34 weeks (platelet 37), no response, then dex 40 mg × 4 days, and IVIG, + 2u of platelets at delivery	20	35 weeks
13	2	38 weeks +4 days	31		Yes (platelets > 70)	No	No	66	34 weeks +4 days
13	1	39 weeks +6 days	33		Yes (platelets > 70)	No	No	55	Post‐partum due to PPH
14	1	35 weeks +3 days	30		Yes (platelets > 70)	Yes, antepartum	IVIG at 18 weeks, and then repeat IVIG at delivery	31	18 weeks
15	1	38 weeks +5 days	33		Yes (platelets > 70)	No	No	99	38 weeks +5 days
16	1	33 weeks +2 days	26	Severe PPH	No	Yes, when admitted for delivery	Dex 40 mg × 4 days, IVIG × 2 doses (at delivery)	39	33 weeks +2 days
17	1	39 weeks +3 days	43		Yes (platelets > 70)	No	No	78	39 weeks +2 days
18	1	39 weeks +1 day	38		Yes (platelets > 70)	Yes, antepartum	Pred 40 mg × 7 days at 12 weeks GA, then IVIG x1	17	12 weeks gestation
19	1	39 weeks +2 days	30		Yes (platelets > 70)	No	No	70	39 weeks 1 day
20	1	39 weeks +1 day	28		Yes (platelets > 70)	No	No	90	15 weeks +1 day
21	1	38 weeks +6 days	28		Yes (platelets > 70)	Yes	No	73	38 weeks +6 days
22	1	37 weeks +6 days	28		Yes (platelets > 70)	No	Pred 60 mg × 3 days, 40 mg × 3 days, 20 mg × 3 days, 10 mg × 3 days, started postpartum for epidural removal	60	37 weeks +6 days
23	1	37 weeks +1 day	32	Pre‐eclampsia	Yes (platelets > 70)	No (maybe outside but nothing in UU system)	Pred (60, 40, 10 mg), then 1u platelet, more pred post‐partum	62	37 weeks
24	1	37 weeks +1 day	21		Yes (platelets > 70)	Yes, antepartum	Pred 60 mg qD × 7 days (at 34 weeks GA), 40 mg × 7 days, 20 mg × 7 days	65	36 weeks
25	1	35 weeks +6 days	24		Yes (platelets > 70)	No	No	96	34 weeks +5 days
26	1	38 weeks +1 day	29		No (platelets > 70)	Yes, antepartum	Pred 1 mg/kg at 35 weeks GA, and then IVIG x2, no improvement, dexa 40 mg × 4 days, repeated IVIG + dexa with good response	6	35 weeks gestation
27	1	37 weeks +6 days	29		Yes (platelets > 70)	No	Started prednisone 50 mg daily at 37 weeks gestation	63	37 weeks gestation
28	1	39 weeks +4 days	26		No	Yes, antepartum	Started prednisone 50 mg BID at 37 weeks, no response	49	23 weeks gestation
29	1	39 weeks +2 days	30		No (platelets > 70)	No	None	69	31 weeks gestation
29	2	40 weeks +4 days	24		No (platelets > 70)	No	No	78	40 weeks +4 days
30	1	39 week s + 5 days	32		Yes (platelets > 70)	No	No	82	39 weeks +5 days
31	1	39 weeks +1 day	27		Yes (platelets > 70)	Yes, antepartum	Pred 20 mg BID at 34 weeks GA, continue throughout post‐partum taper (20 mg qAM, 15 mg qPM x3 days > 15 mg BID x3 days > 10 mg BID x3 days > 5 mg BID x3 days > 5 mg daily x3 days>OFF)	62	18 weeks gestation
32	1	37 weeks +3 days	28		Yes (platelets > 70)	Yes, antepartum	No	81	37 weeks +4 days
33	1	35 weeks +6 days	19	Possibly drug induced ITP	No (platelets > 70)	Yes, post‐partum	No	92	35 weeks

Abbreviations: Dex = dexamethasone, pred = prednisone.

## 4 Discussion

In this cohort of more than 300 pregnancies complicated by moderate‐to‐severe thrombocytopenia, neuraxial anesthesia was performed in 78% of cases—including 71% of those with ITP—with near‐universal adherence to the Society for Obstetric Anesthesia and Perinatology (SOAP)–recommended platelet threshold of ≥ 70 × 10^9^/L [5]. These findings provide real‐world outcomes data supporting current guideline recommendations in a higher‐risk population with thrombocytopenia.

Unlike broader cohorts that included mild thrombocytopenia, our analysis focused exclusively on platelet counts < 100 × 10^9^/L—a group at greater theoretical risk for neuraxial bleeding and for whom management uncertainty is most pronounced [5]. Within this range, institutional practice demonstrated consistent alignment with SOAP guidance and reassuring procedural safety across diverse etiologies, including ITP and HELLP syndrome.

Gestational thrombocytopenia remained the most common etiology even within this severity range and was associated with higher platelet nadirs and later gestational age at delivery, similar to prior reports [7, 8, 9]. ITP comprised 11% of cases, consistent with prior retrospective reports of moderate to severe thrombocytopenia [10, 11]. Among individuals with ITP, hematology consultation and treatment intensity closely tracked thrombocytopenia severity, suggesting risk‐aligned escalation of care. Although our study was not designed to assess the causal effects of consultation, these patterns support multidisciplinary management in higher‐risk presentations of thrombocytopenia.

In our cohort, 48.6% of individuals with ITP received treatment, most frequently corticosteroids alone, and 9% required platelet transfusion—comparable to the 10% transfusion rate reported in a systematic review [12]. Our sample size was insufficient to determine whether hematology consultation reduced transfusion use, an important area for future investigation given the risks associated with platelet transfusion in pregnancy [13]. No individuals in our cohort received thrombopoietin receptor agonists, rituximab, or anti‐D antepartum.

The question of neuraxial anesthesia safety at platelet counts between 50–69 × 10^9^/L remains an area of clinical uncertainty [14, 15, 16]. The current SOAP guidelines state that risk/benefit discussions can guide management at platelet counts between 50–69 × 10^9^/L and that neuraxial anesthesia placement is not unreasonable in certain circumstances [5]. We, however, did not report any neuraxial anesthesia placement within this range, similar to previous reports. In a meta‐analysis of 26 studies, about 57% of ITP patients underwent neuraxial procedures, and clinicians frequently avoided placement with platelet count below 80 × 10^9^/L [17]. In our cohort, individuals with incomplete treatment responses frequently fell within this range and often did not receive neuraxial anesthesia, reflecting cautious adherence to current recommendations. We postulate that early hematology and anesthesia consultation, as recommended by SOAP, may allow for earlier intervention for moderate‐to‐severe thrombocytopenia, for risk/benefit discussions, and the consideration of neuraxial anesthesia within this platelet count range. Implementing a clinical decision pathway to guide consultation of specialists might be warranted in the obstetric setting.

A clinical decision pathway might also help to address the relatively high rate of cesarean deliveries (about 14% higher than the national average), and general anesthesia use among this high‐risk cohort, albeit at an academic medical center with more high‐risk pregnancies [18]. Implementation of this process would ideally prompt earlier hematology and obstetric anesthesia review for patients with suspected ITP, platelet counts < 70 × 10^9^/L, rapidly declining platelet counts, concurrent anticoagulation, or anticipated operative delivery. Such a pathway may allow more timely risk–benefit discussions, treatment planning when appropriate, and clearer documentation of patient preferences regarding neuraxial analgesia.

Currently, at our institution, there is no formal clinical decision pathway for triggering obstetric anesthesia consultation for high‐risk pregnant individuals with a hematologic disorder. We plan to implement a quality improvement initiative where specific criteria (such as history of ITP, moderate‐to‐severe thrombocytopenia in the third trimester, antepartum use of steroids for ITP, etc.) will trigger antepartum obstetric anesthesia consultation. We plan to then compare pre‐ and post‐implementation neuraxial anesthesia rates in this subgroup.

In parallel, a better biologic characterization of bleeding risk in thrombocytopenia states may help guide platelet thresholds. Platelet function varies across thrombocytopenic states, and qualitative platelet integrity in ITP may differ from that seen in preeclampsia, HELLP syndrome, or consumptive coagulopathies [19, 20, 21, 22]. Emerging data using point‐of‐care hemostatic assays, such as thromboelastography, demonstrate that clot strength is preserved even at low platelet counts, suggesting quantity does not necessarily impact clotting quality [23]. Although platelet function testing did not influence anesthetic decision‐making in our cohort, incorporation of functional assessments into prospective studies may help individualize care and provide information in a more timely manner than typical laboratory tests.

This study is limited by its retrospective, single‐center design and modest sample size, which precludes causal inference and limits generalizability to non‐tertiary settings. We were unable to determine whether hematology involvement directly altered neuraxial eligibility or clinical outcomes. Nonetheless, the cohort size and focus on moderate‐to‐severe thrombocytopenia provide meaningful contemporary outcomes data in a population for whom evidence remains sparse. Importantly, neuraxial bleeding risk is multifactorial, and platelet count alone does not fully capture procedural bleeding risk. Many individual‐level factors (e.g., renal function, obesity, etc.) as well as procedural factors modify bleeding risk and influence neuraxial decision‐making independently of platelet count. A limited number of these risk factors could be systematically abstracted for all deliveries, limiting our ability to assess their contribution to neuraxial decision‐making or bleeding outcomes. As no neuraxial hematomas occurred, the study also could not identify independent predictors of neuraxial bleeding. Additionally, given the scope of our paper, we did not examine maternal hemorrhagic and neonatal outcomes; however, this is an important future direction to explore. Finally, our study was limited by a lack of documentation to determine if a pregnant individual's desire for epidural was considered during the risk–benefit discussion for epidural placement, especially for those with a platelet count between 50 and 70 × 10^9^/L. We believe that implementing the clinical management pathway described above would improve documentation consistency, as high‐risk pregnant individuals would receive early anesthesia consultation, during which anesthesia preferences could be discussed and clearly documented.

## 5 Conclusions

Across more than 300 pregnancies with moderate‐to‐severe thrombocytopenia, we observed clear diagnostic, biologic, and management gradients. gTCP was the most common etiology and demonstrated substantially higher platelet nadirs and term gestational delivery compared with ITP and HELLP syndrome. Neuraxial anesthesia was achieved in nearly 80% of pregnancies overall, with strict adherence to institutional platelet thresholds and no neuraxial bleeding complications observed. Hematology consultation and treatment intensity closely tracked platelet severity within the ITP subgroup, suggesting risk‐aligned escalation of care. Together, these findings highlight consistent real‐world guideline implementation and reassuring procedural safety across a diverse thrombocytopenic pregnancy population.

## Funding

The authors have nothing to report.

## Ethics Statement

This study was reviewed and deemed exempt by the University of Utah Institutional Review Board (IRB) under Category 4, as it involved retrospective analysis of existing clinical data with minimal risk to participants.

## Conflicts of Interest

The authors declare no conflicts of interest.

## Supporting information


**Figure S1:** Flowchart of the cohort between January 1, 2018 and December 31, 2022. Of 22 496 pregnancies, 306 met criteria for inclusion in this study.

**Table S1:** ICD‐10 codes used to identify live birth or fetal death (≥ 20 weeks of gestation).

**Table S2:** Delivery route and anesthesia characteristics among pregnancies with moderate‐to‐severe thrombocytopenia. The table summarizes delivery route and anesthesia/analgesia approaches among the 306 pregnancy encounters included in the cohort. Cesarean delivery occurred in 143 pregnancies, with cesarean urgency classified as scheduled, urgent, or emergent. Vaginal delivery and assisted vaginal delivery were grouped together for analysis. Among vaginal deliveries, labor analgesia was categorized as neuraxial anesthesia, no analgesia documented, IV analgesia, or nitrous oxide. Among cesarean deliveries, anesthesia was categorized as neuraxial anesthesia and/or general anesthesia. Values are presented as *n*/*N* (%), with denominators reflecting the relevant delivery subgroup. Emergent cesarean delivery was defined as delivery within 30 min of decision, and urgent cesarean delivery was defined as delivery within 30–75 min of decision. Four cesarean deliveries involved both neuraxial and general anesthesia, so cesarean anesthesia categories are not mutually exclusive.

## Data Availability

The data that support the findings of this study are available from the corresponding author upon reasonable request.
